# TSWV Infection Differentially Reshapes the Symbiotic Microbiome of Two *Frankliniella* Thrips Species

**DOI:** 10.3390/v17121625

**Published:** 2025-12-16

**Authors:** Eeshita Mandal, Nuttapol Noirungsee, Terd Disayathanoowat, Eui-Joon Kil

**Affiliations:** 1 Department of Plant Medicals, Gyeongkuk National University, Andong 36720, Republic of Korea; eeshitamandal1606@gmail.com; 2 Department of Entomology, Faculty of Agriculture, Habiganj Agricultural University, Habiganj 3300, Bangladesh; 3 Department of Biology, Faculty of Science, Chiang Mai University, Chiang Mai 50200, Thailand; nuttapol.n@cmu.ac.th; 4 Research Center of Deep Technology in Beekeeping and Bee Products for Sustainable Development Goals (SMART BEE SDGs), Chiang Mai University, Chiang Mai 50200, Thailand

**Keywords:** *F. occidentalis*, *F. intonsa*, metagenome, symbionts, tospovirus

## Abstract

Vectoring tomato spotted wilt virus (TSWV) by two well-known thrips species, *Frankliniella occidentalis* Pergande and *F. intonsa* Trybom (Thysanoptera: Thripidae), is facilitated in different ways. Symbiotic bacteria positively influence thrips fitness, but the interaction between these bacteria and tospovirus inside the thrips’ body remains unknown. Metagenomic profiling of symbionts in nonviruliferous and viruliferous *Frankliniella* thrips was performed to elucidate the interactions between symbiotic bacteria and the virus. A total of 97 operational taxonomic units (OTUs) were identified by profiling the microbes, where Proteobacteria was the most abundant phylum, with a high richness in *Serratia* spp. *F. occidentalis* showed lower variation in bacterial diversity between nonviruliferous and viruliferous treatments than *F. intonsa*. RT-qPCR validation for *Serratia* and *Escherichia* revealed opposite abundance patterns between the two thrips species. In contrast, Enterobacteriaceae and *Pantoea* showed similar patterns with higher abundance in nonviruliferous conditions. *Wolbachia* was detected exclusively in *F. intonsa*, with a higher bacterial titer in the viruliferous sample. Our findings suggest that TSWV association may influence the abundance of different bacterial symbionts within the thrips’ body, potentially via induction of antimicrobial peptides in response to viral invasion, and to our knowledge this is the first report addressing this tripartite interaction. These findings improve our understanding of how virus–symbiont association contributes to thrips vector competence.

## 1. Introduction

Both *Frankliniella occidentalis* Pergande and *F. intonsa* Trybom (Thysanoptera: Thripidae) are herbivorous in nature and vector a wide range of tospoviruses [1,2]. These two species share similar morphology, feeding behavior, host plant preferences, and ecological niches [3,4,5]. They reduce the productivity of host plants by directly sucking sap and by laying eggs inside plant tissue [6,7]. However, these two thrips species exhibit different biological characteristics and pest statuses; *F. intonsa* has higher fecundity than *F. occidentalis* in hot pepper fields [8], and they show different abundance depending on geographical location [9]. Tomato spotted wilt virus (TSWV) is an important tospovirus reported to be acquired and circulated through the gut of the thrips in a persistent propagative manner [10]. Only the first instar larva can acquire the virus by feeding on the previously infected plant tissue [11]. TSWV was reported to facilitate thrips’ herbivory by modulating the metabolic and plant defense pathways [12].

Symbiotic microbiomes encompass the entire population of microorganisms residing on or within the body of the host organism and are predominantly synergistic. Bacteria may be hosted inside insect cells or in an extracellular tissue. Both types of bacteria are maternally inheritable from generation to generation, which reveals their motility as a component of insect speciation and plant–herbivore competition [13]. The gut microbes are remarkably represented as endosymbionts of the arthropod groups, especially herbivorous insects, and any disturbance to this community may cause fitness loss in the host insect [14]. They facilitate insect adaptation by helping in biomass degradation, nutrition acquisition, detoxification, and other environmental adaptations [15]. Gut bacterial diversity is determined by the habitat, diet, developmental stage, and phylogeny of host insects [16,17]. Thrips host different types of bacteria in their gut and Malpighian tubules [18], which include Enterobacteriaceae and several other species [19]. Metagenomic analysis for *F. occidentalis* by Chanbusarakum & Ullman [20] revealed the presence of *Erwinia* sp. in the thrips gut, which is also a close relative of the Enterobacteriaceae group [21]. However, the symbiotic microbes of *F. intonsa* are still unknown. Additionally, the effect of TSWV association (viruliferous status) on the abundance of bacterial symbionts should be examined, as these two species of thrips are responsible for spreading different plant viruses to their hosts, resulting in significant economic losses [22,23,24,25,26]. However, the symbiotic microbes of *F. intonsa* are still unknown, and it also remains unclear how TSWV association reshapes the microbiomes of key vector species. Comparing the microbiomes of *F. occidentalis* and *F. intonsa* under TSWV association is, therefore, important for understanding species-specific vector competence and for explaining their contrasting pest statuses and virus transmission efficiency in the field.

Gut microbes enhance internal fitness in host insects, whereas viruses modulate external feeding behaviors. The positive interaction of both organisms within the host body may provide strong vector competence. Based on previous work showing species-specific and population-specific microbiomes in thrips and other hemipteran vectors [20], we hypothesized that TSWV association would be accompanied by distinct rearrangements of the bacterial community in *F. occidentalis* and *F. intonsa*. The objectives of this study were (i) to characterize and compare the bacterial symbionts communities associated with nonviruliferous and TSWV-associated *F. occidentalis* and *F. intonsa* and (ii) to evaluate how TSWV association is linked with shifts in key symbionts in these two vector species.

## 2. Materials and Methods

### 2.1. Thrips Rearing

*F. occidentalis* and *F. intonsa* were reared in the Insect Molecular Physiology Laboratory of Gyeongkuk National University, Korea, in a controlled environment of 26 °C, (16:8) hours light and dark period, and 70% relative humidity. The thrips were kept in circular breeding boxes (100 mm × 40 mm, SPL, Pocheon, Republic of Korea) separated according to their life stages: eggs, larvae (L1 and L2), pupae, and adults. Red kidney beans (*Phaseolus vulgaris* Linnaeus) were sown in trays and harvested immediately after sprouting and splitting out of the cotyledon, rupturing the seed coat. Then, those cotyledons were used to feed the thrips of all stages. The diet was replaced daily, and food waste from adult boxes was used to make egg boxes, as they laid eggs inside the plant tissue. It takes 6 to 7 days to complete their transition from larva to adult, with a complete life span of approximately 25 days at a temperature of 26 °C.

### 2.2. TSWV Treatment to Thrips

The first instar larvae (L1) of two thrips species were selected for feeding TSWV, as the L1 stage is eligible for TSWV acquisition by thrips. Fresh hot pepper (*Capsicum annuum* Linnaeus) leaf with identical concentric ringspot symptoms of TSWV was collected from the field and then tested to confirm the presence of virus with the Immunostrip TSWV kit (Agdia, Elkhart, IN, USA). A small portion (about 6 g) of a leaf was ground and diluted with 0.01 M NaOH solution. Previously grown kidney bean cotyledons were kept in the virus-carrying leaf extract for 15 min, then dried for a few minutes in an aseptic environment. Those beans were then fed to the L1 larvae for 24 h and replaced with fresh, uncontaminated beans. A random sample of 30 individuals per replicate was taken from the TSWV-fed insect colony to assess the proportion of viruliferous thrips. RNA was extracted separately from individual insects, and RT-PCR targeting the TSWV N gene was performed followed by gel electrophoresis. Across three biological replicates, on average, approximately 90% of assayed insects were positive for the TSWV gene.

### 2.3. DNA Extraction

There were four selected treatment groups in total: nonviruliferous *F. occidentalis*, viruliferous *F. occidentalis*, nonviruliferous *F. intonsa*, and viruliferous *F. intonsa*. For each treatment, three biological replicates were prepared, each consisting of a pool of approximately 30 adult thrips collected into a 1.5 mL centrifuge tube (Axygen, Union City, CA, USA) without sexing and without any body surface sterilization. Total DNA was extracted using the Invitrogen PureLink Genomic DNA Mini Kit by Thermo Fisher Scientific, Waltham, MA, USA and the DNA concentration and purity were examined using a NanoPhotometer^®^ NP80 (IMPLEN, Munich, Germany). It was then stored at −20 °C until further use. Because whole adults were processed without surface sterilization, the resulting profiles represent the bacterial communities associated with the entire insect body under controlled rearing conditions and may include a minor contribution of external or cuticular bacteria.

### 2.4. Library Preparation and Sequencing

The quality control (QC) criteria for DNA referred to the specification requirements of a single run, which, after passing, proceeded with the library construction. The QC was ensured using a 2100 Bioanalyzer (Agilent Technologies, Santa Clara, CA, USA), and the quantity of DNA was assessed by the PicoGreen (P7589, Invitrogen, Carlsbad, CA, USA) method using Victor 3 fluorometry. The samples were prepared following the quality requirements for NGS library preparation. 16S rRNA amplicon metagenomic sequencing was performed using the Herculase II Fusion DNA Polymerase Nextera XT Index V2 library kit. The library protocol involved 16S rRNA metagenomic sequencing library preparation (Part # 15044223 Rev. B) followed by paired-end sequencing with an amplicon size of 465 bp using Illumina NovaSeq 6000 (Illumina, San Diego, CA, USA) SBS technology. The binary files were then converted to FASTQ files using bcl2fastq conversion software V2.20.

### 2.5. Operational Taxonomic Unit (OTU) Analysis

The raw fastq data were uploaded as a BioProject named ‘Metagenomic sequencing of *Frankliniella*’ in NCBI database, containing the project number PRJNA1031338. The CLC Genomic Workbench 22.0.1 (QIAGEN, Hilden, Germany) was used for analyzing sequenced data. Trimming of the raw reads from each sample was performed first to obtain ‘Trim reads’. The genome of *F. occidentalis* (GenBank: GCF_000697945.2) was used as the reference for mapping thrips-derived reads. Then, the 16S rRNA information of endosymbionts registered in the NCBI database was also collected to map with the previously unmapped reads by custom filtering. The sequence of the OTUs was obtained for the targeted microbes. UPGMA (Unweighted Pair Group Method with Arithmetic mean) clustering was performed using the obtained OTUs to profile the bacteria in each treatment group. An alpha diversity plot was generated using phylogeny, applying rarefaction by iterative subsampling without replacement with a sequencing depth of 40,000, and a 3D PCoA plot was generated through Principal Coordinates Analysis (PCoA) by using unweighted UniFrac values.

### 2.6. Making Phylogenetic Tree for Pairwise Comparison

A phylogenetic tree was constructed using the sequences for bacterial OTUs with MEGA 11 software (version 11.0.13), comparing them with the complete sequences of each bacterium from the NCBI GenBank. The maximum likelihood method was used for the tree, and 1000 bootstrap iterations were used for nucleotide distance measurement [27,28].

### 2.7. RT-qPCR

Specific bacterial sequences identified in the analyzed data were selected for quantitative analysis using RT-qPCR, targeting the selected genes from biological replicates. Primer designing was performed using the NCBI Primer-BLAST tool (https://www.ncbi.nlm.nih.gov/tools/primer-blast/ [accessed on 4 September 2023]). First, the bacteria were confirmed by a 20 µL PCR reaction at different temperature cycles using gene-specific primers (Supplementary Table S1) and AmpONE *Taq* DNA polymerase (GeneAll, Seoul, Republic of Korea). Electrophoresis for confirmation of the PCR product was performed using 1% agarose gel in Mupid^®^-2plus (Takara Bio Inc., Shiga, Japan) for 25 min. The size marker used for comparing the product size was a 100 bp DNA ladder (Bioneer, Daejeon, Republic of Korea). Gel checking was performed using GelDoc Go (Bio-Rad, Hercules, CA, USA). In case of qPCR, SYBR Green PCR Master Mix (Thermo Fisher Scientific) was used, and Elongation factor 1 (EF1) was used as the housekeeping primer to compare C_T_ (2^−ΔΔCT^) values [29].

### 2.8. Molecular Interaction Analysis

The molecular mechanisms underlying the interaction between TSWV and symbiotic bacteria were explored. A pathway was made showing how the invasion of TSWV inside a thrips body influences the abundance of symbiotic bacteria. The pathways were considered in accordance with the typical pathways for insect innate immune signaling, as described by Bangham et al. [30]. The abundance of antimicrobial peptides in response to TSWV infection, as reported by Mandal et al. [31], is compiled in a table that is effective against both the virus and bacteria.

### 2.9. Statistical Analysis

Statistical analyses, including ANOVA and the LSD mean comparison test, were performed using the SAS 9.4 program [32].

## 3. Results

### 3.1. Sequencing Summary

The 16S rRNA metagenomic sequencing of *F. occidentalis* and *F. intonsa* by Illumina gave variable reads for nonviruliferous and viruliferous treatments. The highest number of raw reads was obtained from nonviruliferous *F. intonsa*, whereas the lowest was from viruliferous *F. intonsa*. After filtering reads in OTU, the number ranged between 41,341 and 14,070; meanwhile, viruliferous *F. occidentalis* showed the highest percentage of data merge (34.24%) (Supplementary Table S2). There was an average of 70.00% unmerged data for the overall treatment groups.

### 3.2. OTU Analysis and Microbiome Mapping

**Figure 1:**
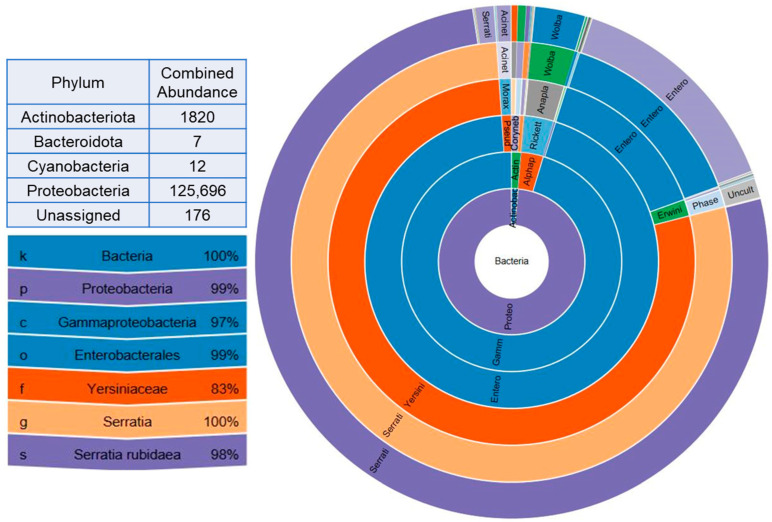
Combined abundance summary of operational taxonomic units (OTUs) from nonviruliferous and viruliferous treatment groups of *Frankliniella occidentalis* and *F. intonsa*. Total abundance represented at the phylum level with distribution of core microbial community at different taxonomic levels (Kingdom, phylum, class, order, family, genus, species).

A total of 97 OTUs were obtained from 16S rRNA metagenomic analysis of four different treatments in two thrips species. Four different phyla were represented by the total number of bacterial endosymbionts obtained from the analysis, among which Proteobacteria were the most abundant, with an unassigned group accounting for 0.14% of the individuals. *Serratia rubidaea* represented the core microbiota, accounting for 98% of the total abundance, indicating dominance among the symbionts (Figure 1). 

**Figure 2:**
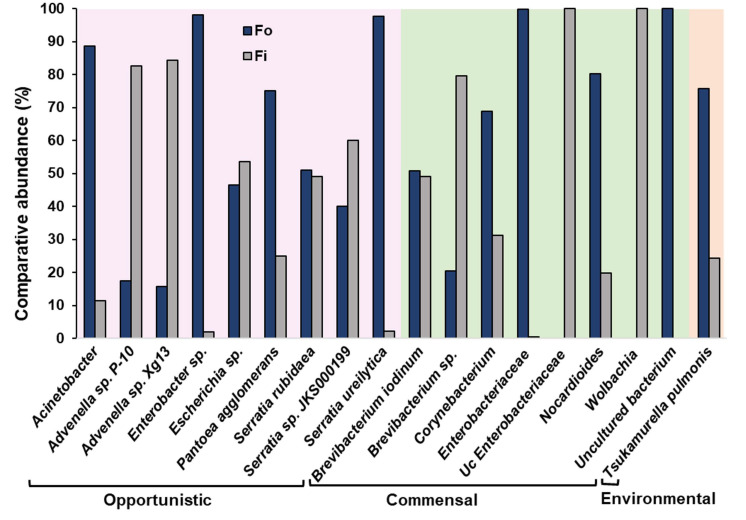
Comparative abundance of selected symbiotic bacterial species between two different thrips species obtained by metagenomic analyses grouped according to their functional nature. The total value for each bar group ranges from 0 to 100 percent (Fo, *Frankliniella occidentalis*; Fi, *F. intonsa*).

Comparison of the percentage of selected individual bacteria between the two thrips species showed highly variable results. Uncultured Enterobacteriaceae bacterium (GenBank accession: JN793838) and *Wolbachia* endosymbiont of *Culex quinquefasciatus* (GenBank accession: AM999887) were abundantly present in *F. intonsa* but absent in *F. occidentalis*. It was the first case to identify *Wolbachia* in *F. intonsa*. Further sequencing of the PCR product was confirmed and showed 98% similarity with the NCBI database sequence for *Wolbachia* endosymbiont of *C. quinquefasciatus* (Supplementary Figure S1). In contrast, uncultured bacteria (GenBank accession: JF121027, JF830198) were present only in *F. occidentalis*. *Pantoea agglomerans* (GenBank accession: AB004691) was higher in *F. occidentalis*, and *S. rubidaea* (GenBank accession: AB004751, KJ865575) was nearly similar in both thrips species (Figure 2).

**Figure 3:**
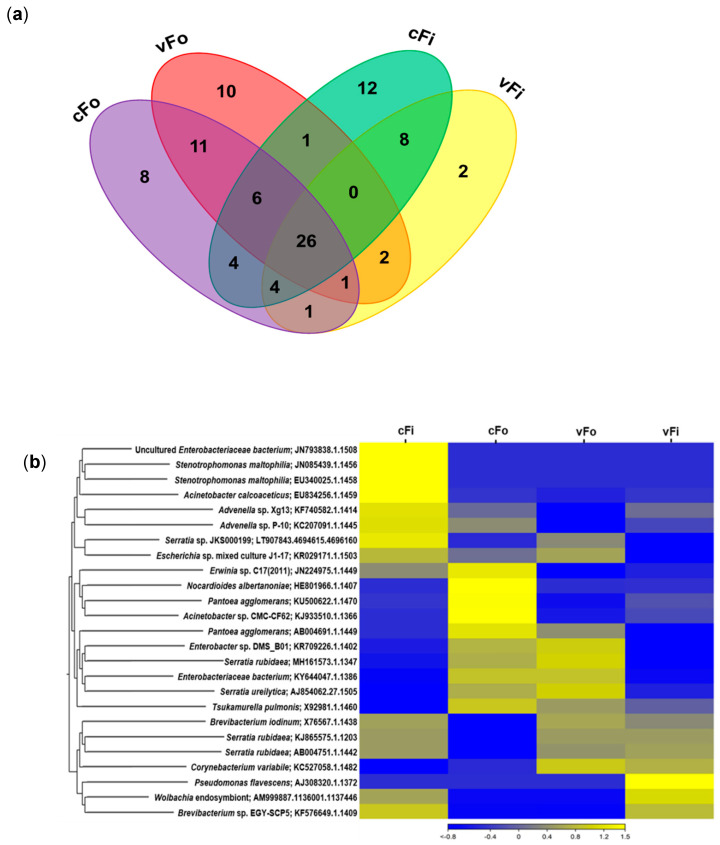
Microbiome mapping in different treatment groups of two thrips species. (**a**) Venn diagram showing the overlap of OTUs among the treatments; (**b**) heatmap clustering comparing the abundance of different bacteria among different treatments (cFi, nonviruliferous *Frankliniella intonsa*; cFo, nonviruliferous *F. occidentalis*; vFo, viruliferous *F. occidentalis*; vFi, viruliferous *F. intonsa*).

Among the 97 OTUs, 26 were common to all treatment groups, while the uniquely present OTUs differed for each (cFo = 8, vFo = 10, cFi = 12, vFi = 2) (Figure 3a). A heatmap clustering analysis was conducted to compare relative abundances of selected bacterial OTUs among treatments. Nonviruliferous *F. intonsa* showed an enrichment in uncultured Enterobacteriaceae bacterium, *Stenotrophomonas maltophilia*, and *Acinetobacter calcoaceticus*, while viruliferous *F. intonsa* showed unique enrichment in *Pseudomonas flavescens*. Nonviruliferous *F. occidentalis* was enriched in *Nocardioides albertanoniae*, *P. agglomerans*, and *Acinetobacter* sp. Similarity in clustering was found mostly between viruliferous and nonviruliferous *F. occidentalis* and a little in viruliferous and nonviruliferous *F. intonsa* (Figure 3b).

**Figure 4:**
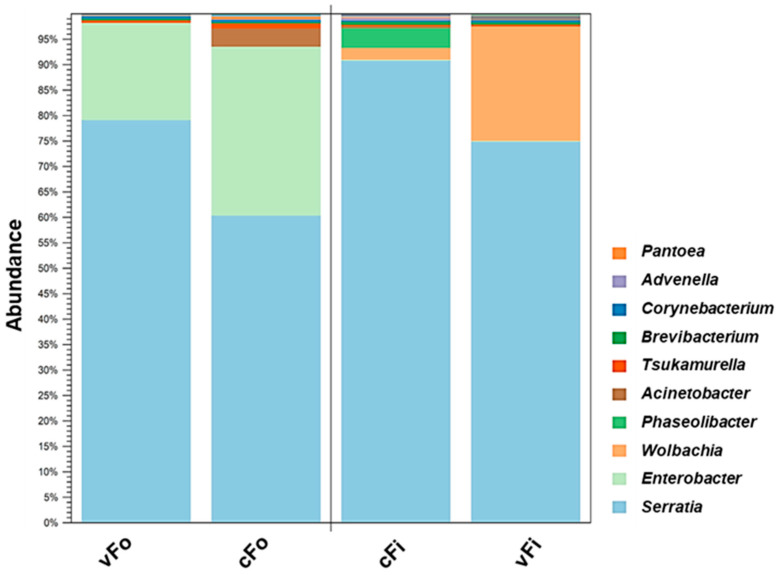
Abundance of bacterial 16S rRNA gene sequences at the genus level with UPGMA clustering in two thrips species after TSWV infection. Bacterial genera are indicated with different colors arranged by placing the most abundant at the bottom (UPGMA = Unweighted Pair Group Method with Arithmetic mean; vFo, viruliferous *Frankliniella occidentalis*; cFo, nonviruliferous *F. occidentalis*; cFi, nonviruliferous *F. intonsa*; vFi, viruliferous *F. intonsa*).

Clustering analysis among four treatments for 10 highly abundant bacterial genera showed the highest percentage for *Serratia* (77.79%) within the total bacterial community. Specifically, it was highest in nonviruliferous *F. intonsa* (90.84%) and lowest in nonviruliferous *F. occidentalis* (60.39%), compared to all other genera presented. *Enterobacter* was the second most abundant (14.21%) and was commonly present in nonviruliferous *F. occidentalis* (32.78%); it was less abundant in viruliferous *F. intonsa* (0.11%). *Wolbachia* was the following abundant genus (3.21%) and was present only in *F. intonsa*, with a higher level in the viruliferous treatment (22.49%). Other abundant genera included *Phaseolibacter*, *Actinobacter*, *Tsukamurella*, *Brevibacterium*, *Corynebacterium*, *Advenella*, and *Pantoea*, accordingly (Figure 4).

**Figure 5:**
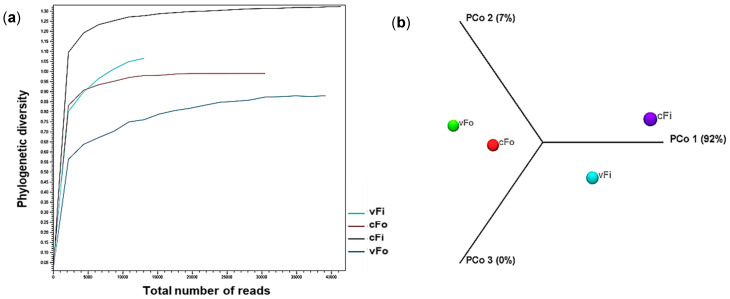
Diversity of bacterial species in two thrips species with TSWV association. (**a**) Alpha diversity plot generated by phylogenetic evaluation; (**b**) 3D PCoA plot generated through Principal Coordinates Analysis (PCoA) by using unweighted UniFrac values.

Also, the microbial community structure across different treatment groups in two thrips species is presented by the Alpha diversity plot (Figure 5a) and the 3D PCoA plot (Figure 5b) to show the species richness and evenness. The highest richness was observed in nonviruliferous *F. intonsa*, while the lowest was found in the viruliferous *F. occidentalis* insect group.

### 3.3. Phylogenetic Analysis and Pairwise Comparison

**Figure 6:**
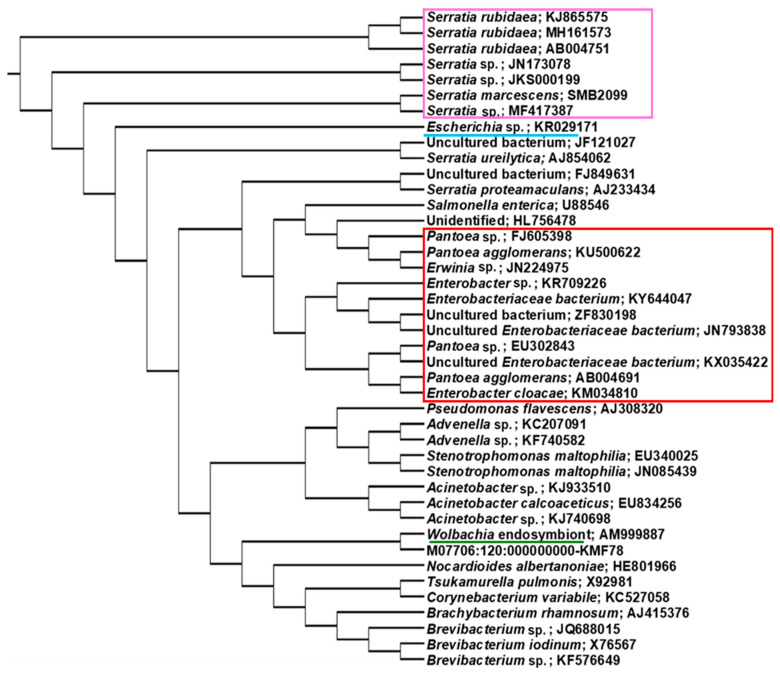
Phylogenetic grouping of observed OTUs in 16S analysis. Bacteria represented in the phylogenetic tree were selected on an abundance basis. Species were separated when there were more than 97% sequence similarity with 16S rRNA sequences available in NCBI GenBank. GenBank accession numbers are added with each species name. Purple box represented the clustering of *Serratia* together and the red box represented the clustering of *Pantoea*, *Erwinia*, and Enterobacteriaceae together.

Ninety-five amplicon sequences of the 16S rDNA of different bacterial species obtained from the four treatments in *F. occidentalis* and *F. intonsa* were used for phylogenetic analysis to compare their taxonomic position, and biological replicates consistently clustered together. Strains of *S. rubidaea* were clustered separately from other *Serratia* species. *Pantoea*, *Erwinia*, and Enterobacteriaceae were grouped together, while *Advenella*, *Wolbachia*, *Tsukamurella*, and *Brevibacterium* formed a different group (Figure 6).

### 3.4. Quantitative Bioassay of Selected Endosymbionts by RT-qPCR

**Figure 7:**
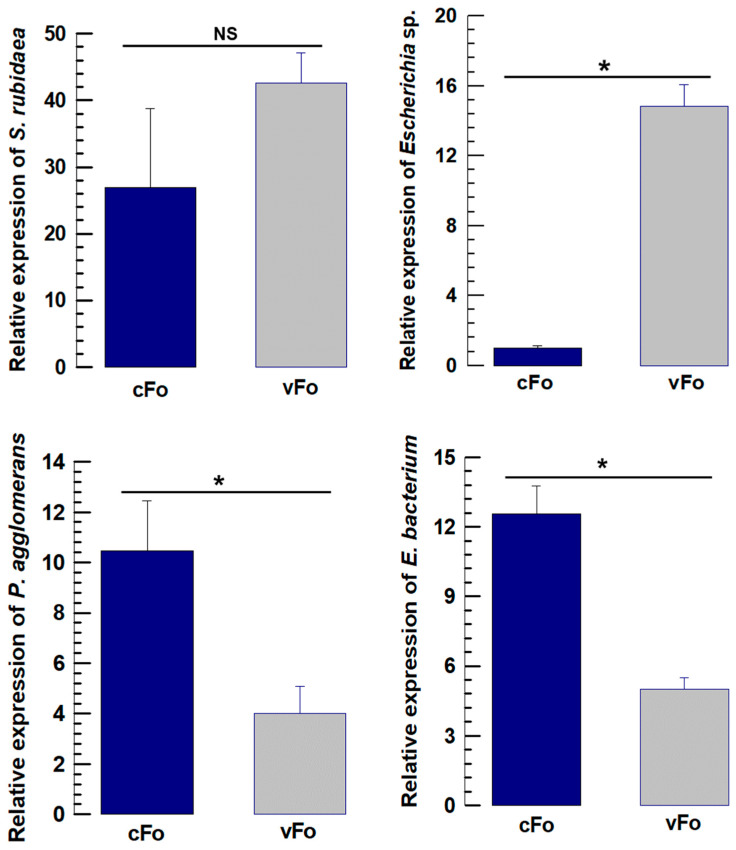
16S rRNA abundance analysis by RT-qPCR in nonviruliferous and viruliferous treatment groups of *Frankliniella occidentalis*. The selected bacteria are *Serratia rubidaea*, *Escherichia* sp., *Pantoea agglomerans*, and Enterobacteriaceae bacterium. The abundance levels expressed as comparative C_T_ (2^−ΔΔCT^) values at different treatments were normalized using the housekeeping gene EF1 (Elongation factor 1). Three different biological replicates were used for each measurement. The asterisk (*) above the bars indicates significant differences among the means at type I error = 0.05, and NS indicates no statistical difference (cFo, nonviruliferous *F. occidentalis*; vFo, viruliferous *F. occidentalis*).

The quantitative bioassay of four selected endosymbionts for *F. occidentalis* revealed a similar abundance pattern to that observed in metagenomic data. *Escherichia* sp. was significantly higher in viruliferous *F. occidentalis* than in nonviruliferous treatment (*F* = 130.57; *df* = 1,4; *p* < 0.001). *P. agglomerans* was significantly higher in the nonviruliferous treatment than in the viruliferous group (*F* = 8.28; *df* = 1,4; *p* = 0.045), which showed a similarity with the abundance pattern of Enterobacteriaceae bacterium (*F* = 34.73; *df* = 1,4; *p* = 0.004) (Figure 7).

**Figure 8:**
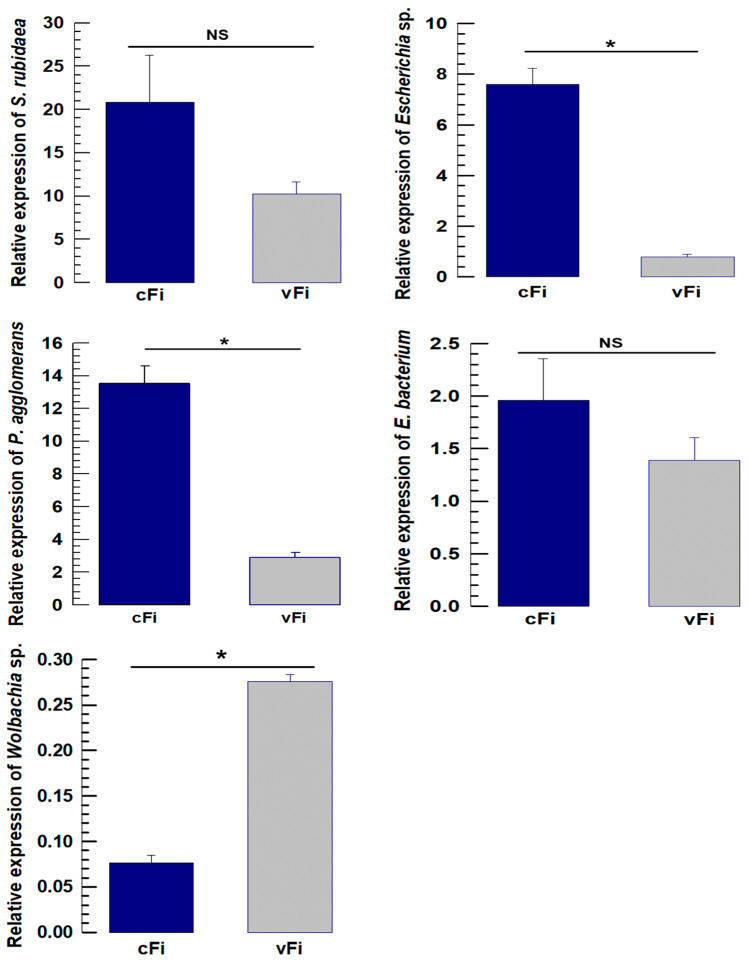
16S rRNA abundance analysis by RT-qPCR in nonviruliferous and viruliferous treatment groups of *Frankliniella intonsa*. The selected bacteria are *Serratia rubidaea*, *Escherichia* sp., *Pantoea agglomerans*, Enterobacteriaceae bacterium, and *Wolbachia* sp. (endosymbiont of *Culex quinquefasciatus*). The abundance levels expressed as comparative C_T_ (2^−ΔΔCT^) values at different treatments were normalized using the housekeeping gene EF1 (Elongation factor 1). Three different biological replicates were used for each measurement. The asterisk (*) above the bars indicates significant differences among the means at type I error = 0.05, and NS indicates no statistical difference (cFi, nonviruliferous *F. intonsa*; vFi, viruliferous *F. intonsa*).

The quantitative bioassay of five selected endosymbionts for *F. intonsa* also showed a similar abundance pattern to the metagenomic data, which differs slightly from that of *F. occidentalis*. *Escherichia* sp. was significantly higher in nonviruliferous *F. intonsa* than in the viruliferous treatment (*F* = 111.28; *df* = 1,4; *p* < 0.001). *P. agglomerans* was also significantly higher in the nonviruliferous treatment than in the viruliferous group (*F* = 90.10; *df* = 1,4; *p* < 0.001), while *Wolbachia* showed an opposite pattern of abundance with a higher abundance in viruliferous *F. intonsa* than in the nonviruliferous group (*F* = 319.05; *df* = 1,4; *p* < 0.001). *S. rubidaea* and Enterobacteriaceae bacterium were not statistically significant in their abundance but were consistent with metagenomic data (Figure 8).

### 3.5. TSWV–Bacteria Association in the Thrips Body

Thrips host both viruses and bacteria, forming a co-association of viral and bacterial communities. The virus influences bacterial abundance in the host body, acting as the most active initiator to ensure that the vector’s fitness is more suitable for virus multiplication by diverting antimicrobial activities towards bacteria rather than the virus [33].

**Figure 9:**
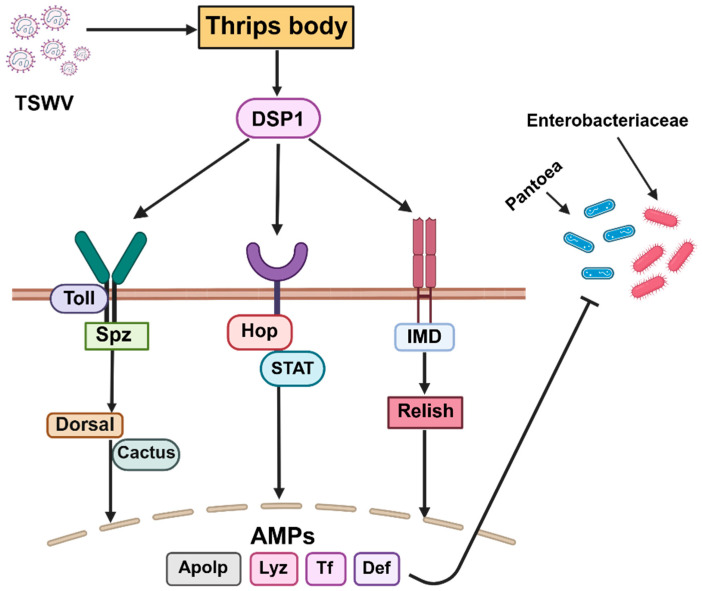
TSWV invasion in thrips influences the abundance of symbiotic bacteria through antimicrobial actions. Dorsal switch protein (DSP1) activates the Toll, JAK/STAT, and IMD signaling pathways. Spätzle (Spz) acts as the ligand for the Toll receptor, while Hop (Hopscotch) represents the insect homolog of Janus kinase (JAK) in the STAT pathway. Arrows indicate activation or induction, whereas blunt-ended lines indicate inhibition. Activation of these pathways leads to the production of antimicrobial effectors including defensin (Def), apolipophorin (Apolp), lysozyme (Lyz), and transferrin (Tf).

After TSWV invasion inside a thrips body, DSP1 (damage-signaling molecule) initiates the systemic immune responses inside the thrips body that activates the Toll, IMD, and JAK/STAT signaling pathways, finally producing antimicrobial peptides. Those AMPs reduce the virus particles as well as bacterial abundance inside the thrips body (Figure 9). TSWV infection significantly increased the expression of Defensin, Transferrin, Apolipophorin, and Lysozyme in *F. thrips* (Supplementary Table S3, data compilation from the study by Mandal et al. [31]), which may negatively influence *Pantoea* and Enterobacteriaceae bacteria abundance.

## 4. Discussion

Insect gut microbiomes are tightly linked to host nutrition, immunity, and adaptation, and their composition is shaped by host phylogeny and ecology [34,35,36,37,38]. In line with this, our metagenomic profiling revealed that both *F. occidentalis* and *F. intonsa* harbored microbiomes dominated by Proteobacteria, with Actinobacteria also represented, similar to previous reports for *T. tabaci* and other thrips species [35,36,37,38]. Both species shared a core set of bacterial taxa but also harbored species-specific symbionts, indicating that closely related thrips occupying similar ecological niches can still maintain distinct microbial signatures. Because adult males and females were pooled within each treatment, potential sex-specific microbiome differences were not evaluated and may contribute to within-treatment variability; however, the consistent clustering of biological replicates suggests that any sex effects did not obscure the main patterns associated with TSWV association and species identity.

The dominance of one particular bacterial species in thrips is a common phenomenon detected in previous studies of *F. occidentalis* [19] and *Frankliniella fusca* Hinds [39]. In our study, *Serratia* was the most abundant genus in both *Frankliniella* species, and *S. rubidaea* constituted the core microbiota in all treatments. The exceptionally high relative abundance of *S. rubidaea* appears to be a specific feature of the populations and rearing conditions examined here, but it is consistent with previous reports of *Serratia*-dominated communities in thrips and other hemipteran vectors. The abundance of *S. rubidaea* was not significantly affected by TSWV association in either species, as further confirmed by RT-qPCR, suggesting that the interaction between *S. rubidaea* and TSWV is largely neutral with respect to abundance. Previously, *Serratia* species have been identified as endosymbionts in *F. occidentalis* and *T. tabaci* [20,40], and *S. symbiotica* has been reported in aphids as a secondary endosymbiont [41,42]. However, this study adds a dimension to the association by exploring the nature of interactions involving the virus within the thrips’ body.

Interactions between *Escherichia* and TSWV differed between thrips species, indicating species-specific microbial responses to viral infections [20]. In *F. occidentalis*, the abundance of *Escherichia* was positively influenced by TSWV, while in *F. intonsa*, it was negatively influenced.

*Pantoea* and *Erwinia* (Enterobacteriaceae) were previously reported to be dominant endobacteria in *F. occidentalis*, *F. intonsa*, and *T. tabaci*, but the viruliferous status of these thrips was not known [20,43,44]. Additionally, among the *Pantoea* species, *P. agglomerans* was detected in OTUs, while other *Pantoea* species remained undetectable, indicating a discrepancy with previously identified *Pantoea* symbionts by Jin & Kim [43]. This may occur due to differences in temperature, habitat, diet, and developmental stages [16,17], as well as viral infections.

This study revealed a lower abundance of *Pantoea* and an uncultured Enterobacteriaceae bacterium in both *Frankliniella* species, particularly in the presence of TSWV infection and high *Serratia* abundance. This supports the fact that microbial interactions influence the microbiome composition revealed by Kozlova et al. [45], finding an inverse correlation between Enterobacteriaceae and *Serratia* in the gut microbiome of *Aedes aegypti*. Again, the transcriptomic data from the study by Mandal et al. [31] showed that TSWV infection significantly upregulated the antimicrobial peptides Defensin and Transferrin in *Frankliniella* thrips, which are the final products of a systemic immune signaling network against a microbial attack (Figure 9). Those AMPs produced by innate immune activity in insects work against bacteria like *Pantoea* [46]. Notably, TSWV cannot significantly activate the IMD signaling pathway in thrips [31], which is considered the primary immune response to bacterial invasion within the insect body.

*Wolbachia* was present only in *F. intonsa* with a higher amount in viruliferous than nonviruliferous, but it was not present in *F. occidentalis* at all, as previously known [19,20,44]. Therefore, the presence of this bacterium had a positive interaction with TSWV association, which is reported here for the first time. *Wolbachia* is important to facilitate reproduction in many insect hosts and has been reported from avocado thrips populations, i.e., *Frankliniella* sp. [47], *H. carpathicus* [37], and *Thrips palmi* Karny [48]. *Wolbachia pipientis*, a common insect endosymbiont, has been reported to reduce mortality induced by a range of RNA viruses [49], which is not the case here; the amount of *Wolbachia* was higher in association with TSWV.

*F. occidentalis* and *F. intonsa* showed significant variation in the amount and types of gut bacteria between nonviruliferous and viruliferous treatment groups. Thus, viruliferous status affects the abundance of different bacterial endosymbionts in two thrips species, either positively or negatively, depending on the bacterial species. Gut bacteria in thrips from other local populations (Hawaii, California, Germany, and The Netherlands) were reported to be unique in their phylogenetic integrity, promoting a symbiotic relationship [20]. However, in Korea, symbiotic bacteria were isolated from *F. occidentalis*, *F. intonsa*, and *T. tabaci* to address the symbiotic role of *Pantoea* in host insect development [43]. This study’s uniqueness lies in its comparison of the endosymbionts of *F. occidentalis* and *F. intonsa* after multiplying the TSWV isolates in their gut. Viruliferous status showed both positive and negative interactions with the gut microbes of two thrips species. Previously, a review of the endosymbiotic bacteria of sweet potato whitefly has explained their biological characteristics, diversity, and their interactions with whitefly-transmitted plant viruses [50]. A GroEL protein homolog from endosymbiotic bacteria of the whitefly (*Bemisia tabaci* Gennadius) is reported to be responsible for the transmission of tomato yellow leaf curl virus [51], and the viruliferous status in aphids (*Myzus persicae* Sultzer) was also reported to reduce the abundance of obligate endosymbiont *Buchnera aphidicola* [52]. These findings collectively highlight that plant virus infection not only affects the physiology of the insect vector but also reshapes its internal microbial ecology, which is essential for understanding the vector fitness.

## 5. Conclusions

In this study, we characterized the bacterial microbiomes associated with nonviruliferous and TSWV-associated *F. occidentalis* and *F. intonsa* and showed that TSWV association is accompanied by species-specific rearrangements of several key symbionts. *Serratia rubidaea* dominated the microbiome of both species, whereas members of Enterobacteriaceae and *Wolbachia* showed contrasting responses to TSWV association between *F. occidentalis* and *F. intonsa*. Together with previously reported transcriptional data on antimicrobial peptide induction in TSWV-infected thrips [31], our results suggest that virus-triggered immune responses may indirectly modulate symbiont abundance in a species-specific manner. These tripartite interactions between virus, vector, and microbiota have clear agricultural implications because symbionts that are positively or negatively associated with TSWV may represent targets for microbiome-based strategies to reduce vector competence and virus transmission in crops. Future work should (i) functionally test the role of dominant and TSWV-responsive symbionts (e.g., *Serratia*, *Pantoea*/Enterobacteriaceae and *Wolbachia*) in virus acquisition, replication, and transmission by experimental manipulation of the microbiome; (ii) assess whether disruption or augmentation of key symbionts can be developed into microbiome-based or symbiont-targeted control strategies to reduce TSWV spread in greenhouse and field crops; and (iii) extend microbiome profiling to additional thrips populations and tospoviruses to evaluate how robust and broadly applicable these patterns are under commercial production conditions. These steps will be essential for translating our molecular and microbiological findings into practical tools for sustainable crop protection.

## Data Availability

The sequencing data generated in this study are openly available in the NCBI Sequence Read Archive (SRA) under BioProject accession number PRJNA1031338. Additional data supporting the findings of this study are included within the article and Supplementary Materials. No other publicly archived datasets were generated.

## Supplementary Materials

The following supporting information can be downloaded at: https://www.mdpi.com/article/10.3390/v17121625/s1, Table S1: Primers used in this study; Table S2: Sequencing summary of metagenomic data in viruliferous and nonviruliferous *Frankliniella occidentalis* and *F. intonsa*; Table S3: Expression of antimicrobial peptides in *Frankliniella occidentalis* and *F. intonsa* retrieved from transcriptome analysis; Figure S1: Confirmation of *Wolbachia* by metagenomic data and validation by PCR, followed by gel electrophoresis.

## Abbreviations

The following abbreviations are used in this manuscript:

IMD	Immune deficiency
JAK/STAT	Janus kinase/signal transducers and activators of transcription
LSD	Least significant difference
OTU	Operational taxonomic unit
PCoA	Principal coordinates analysis
rDNA	Ribosomal DNA
TSWV	Tomato spotted wilt virus
