# Supplementary material for: TSWV Infection Differentially Reshapes the Symbiotic Microbiome of Two Frankliniella Thrips Species
Source: Viruses. 2025 Dec 16;17(12):1625. doi: 10.3390/v17121625 (PMC12737653; doi:10.3390/v17121625)
Supplement: Supplementary file 1 [file viruses-17-01625-s001.zip › viruses-4030312-supplementary.pdf]

Table S1. Primers used in this study

Name	Primer	Product size (bp)
EF-1	5′-TCAAGGAACTGCGTCGTGGAT-3′	130
	5′-ACAGGGGTGTAGCCGTTAGAG-3′	
Serratia	5′-CATGCCGCGTGTGTGAAGAA-3′	184
	5′-TGCGCTTTACGCCCAGTAAT-3′	
Pantoea	5′-CTGCATTCGAAACTGGCAGG-3′	127
	5′-AGCGTCAGTCTTTGTCCAGG-3′	
Enterobacteriaceae	5′-TAATACGGAGGGTGCAAGCG-3′	127
	5′-ACTCTAGCCTGCCAGTTTCG-3′	
Escherichia	5′-GAGGAGGAAGGTGGTGAGC-3′	214
	5′-ACTCTAGCTTGCCAGTTTCAA-3′	
Wolbachia	5′-GGCGCGTAGGCTGGTTAATA-3′	169
	5′-AACCAGATAGACGCCTTCGC-3′	

Table S2. Sequencing summary of metagenomic data in viruliferous and nonviruliferous *Frankliniella occidentalis* and *F. intonsa*

Sample	Treatments	Total number of reads	Filtered reads in OUT [1]	% Merged reads
F. occidentalis	Nonviruliferous	144,578	31,811	27.52
	Viruliferous	141,612	40,489	34.24
F. intonsa	Nonviruliferous	150,404	41,341	33.17
	Viruliferous	76,211	14,070	24.50

[1] Operational Taxonomic Unit.

Table S3. Expression of antimicrobial peptides in *Frankliniella occidentalis* and *F. intonsa* retrieved from transcriptome analysis

**Gene Annotation**	**Gene ID**	**RPKM c*Fo***	**RPKM v*Fo***	**RPKM c*Fi***	**RPKM v*Fi***
Defensin (Def)1	113209991	292.3 ± 34.7	673 ± 70	38.4 ± 18.99	48.45 ± 9.6
Defensin (Def)2	113210584	1.3 ± 0.8	2.98 ± 0.4	0.00	0.00
Apolipophorin (Apolp)1	113210405	508.3 ± 42.1	428.1 ± 61.9	628.4 ± 31.9	659.2 ± 21.8
Apolipophorin (Apolp)2	113204756	7.6 ± 0.8	5.8 ± 1.2	5.4 ± 0.5	2.9 ± 0.34
Lysozyme (Lyz)1	113209799	1.9 ± 0.2	2.9 ± 0.4	0.19 ± 0.1	0.18 ± 0.04
Lysozyme (Lyz)2	113214190	0.17 ± 0.03	0.17 ± 0.02	0.01 ± 0	0.00
Transferrin (Tf)	113213022	4.16 ± 0.4	8.7 ± 1.2	4.16 ± 0.4	2.8 ± 0.34

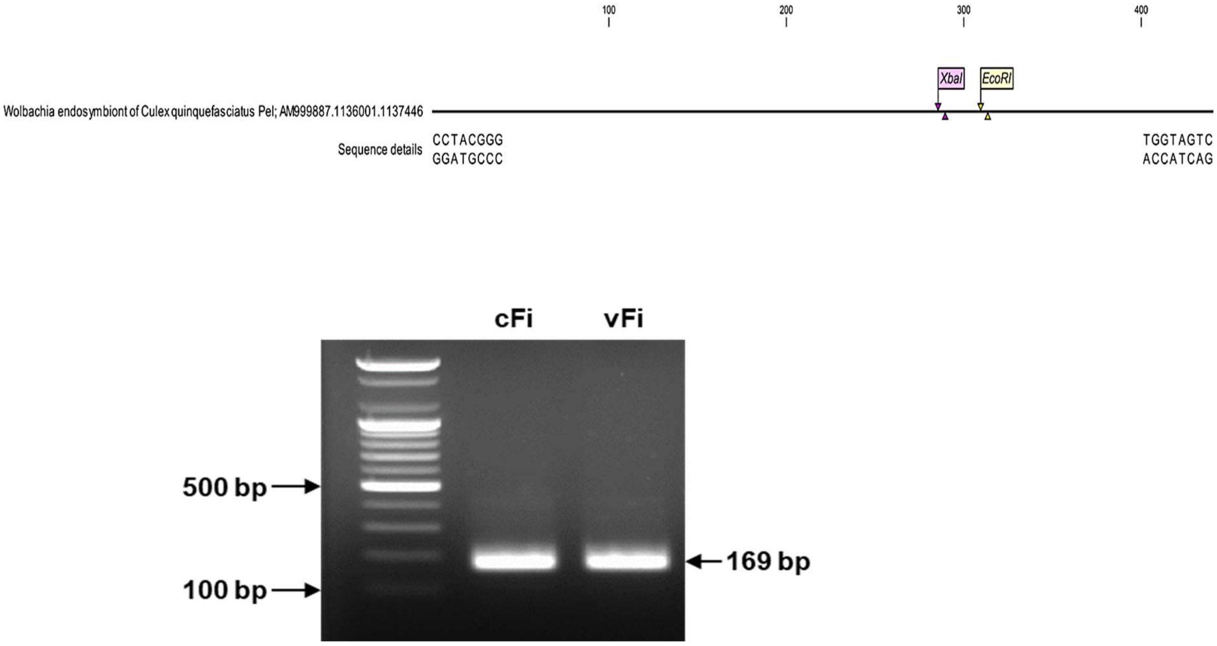

Figure S1. Confirming *Wolbachia* by metagenomic data and re-checking by PCR test, followed by gel electrophoresis.
